# CardioMEMS Heart Failure System: An Up-to-Date Review

**DOI:** 10.7759/cureus.77816

**Published:** 2025-01-22

**Authors:** Oluwaremilekun Tolu-Akinnawo, Naveed Akhtar, Nirav Zalavadia, Maya Guglin

**Affiliations:** 1 Internal Medicine, Meharry Medical College, Nashville, USA; 2 Cardiology, Piedmont Augusta Hospital, Augusta, USA; 3 Internal Medicine, Piedmont Augusta Hospital, Augusta, USA; 4 Advanced Heart Failure, Indiana University School of Medicine, Indianapolis, USA

**Keywords:** cardiomems, device monitoring, heart failure, preventive cardiology, remote monitoring

## Abstract

Heart failure (HF) continues to represent a significant public health concern. While patients with HF with reduced ejection fraction (HFrEF) face a high risk of arrhythmias, both patients with HFrEF and HF with preserved ejection fraction (HFpEF) experience fluid overload, leading to repeated hospitalizations. Traditional monitoring methods have limited ability to manage HF exacerbations preemptively. However, the CardioMEMS™ (Abbott Laboratories, Chicago, IL) HF system, an implantable microelectromechanical sensor, has emerged as an innovative solution, enabling real-time remote monitoring of pulmonary artery pressures (PAPs). CardioMEMS has significantly reduced HF-related hospitalizations and improved patient quality of life (QOL) by facilitating early intervention before clinical symptoms. This article explores the technical specifications, clinical efficacy, integration into clinical practice, and economic impact of the CardioMEMS system. Furthermore, we discuss the challenges and limitations associated with its widespread adoption and propose considerations for future research to enhance its cost-effectiveness and accessibility in diverse healthcare settings.

## Introduction and background

Heart failure (HF) is increasingly recognized as a critical public health issue and is one of the leading causes of hospitalization across the Western world. A significant portion of the affected population has HF with reduced ejection fraction (HFrEF) [1]. Patients with HFrEF face an elevated risk of arrhythmias and struggle with maintaining adequate peripheral perfusion, a challenge that triggers neurohormonal pathways leading to adverse cardiac remodeling and increased afterload, thereby exacerbating HF decompensation [2,3]. The global prevalence of chronic HF is estimated to be between 1% and 2% among the adult population, a figure that is steadily rising due to the aging demographic and advancements in the management of cardiovascular diseases [4-6].

Decompensated HF leads to the progressive deterioration of myocardial function, which not only diminishes the quality of life (QOL) but also imposes a significant economic burden due to increased healthcare costs (hospitalization, emergency department visits, diagnostic tests, and medication prescriptions) associated with HF [7]. Despite remarkable progress in HF management strategies, these patients require vigilant outpatient follow-up and are frequently hospitalized due to episodes of acute decompensation. Statistics reveal that 12-month hospitalization rates range from 32% for ambulatory patients to 44% for those recently hospitalized, underscoring the ongoing challenges in managing this condition [8].

Traditionally, the remote monitoring of HF patients has focused on noninvasive methods, such as tracking symptoms, especially shortness of breath, and physiological parameters including heart rate, weight, and blood pressure. While useful, these methods have limitations in the early detection and proactive management of heart failure exacerbations. Over the past decade, the medical field has witnessed a significant paradigm shift with the advent of microelectromechanical systems (MEMS), which have revolutionized the management of various health conditions, including heart failure. The MEMS technology converts mechanical signals into electrical signals, allowing for more precise and continuous monitoring [9]. This shift was further accelerated by the COVID-19 pandemic, highlighting the need for innovative remote monitoring solutions in healthcare.

In the realm of cardiology, the CardioMEMS™ (Abbott Laboratories, Chicago, IL) device has emerged as a groundbreaking solution, addressing the critical challenges of recurrent exacerbations, high mortality rates, and frequent hospitalizations in heart failure management. The underlying concept of CardioMEMS is based on the observation that hemodynamic congestion (a buildup of pressure in the heart and lungs due to the heart's inability to pump blood efficiently) often precedes the clinical signs and symptoms of HF by several weeks [10]. This insight has paved the way for the proactive management of HF, intending to prevent acute decompensation before it becomes clinically apparent.

The CardioMEMS device was first approved for use in the United States by the Food and Drug Administration (FDA) in 2014. The technology enables real-time, remote monitoring of pulmonary artery pressures (PAPs), which reflect the strain on the heart and lungs caused by fluid buildup, making them an early indicator of heart failure worsening, allowing healthcare providers to adjust medications to keep these pressures within a therapeutic range. This proactive approach can detect early signs of fluid overload, enabling timely intervention before the onset of acute cardiac decompensation. Moreover, since the CardioMEMS system does not rely on battery power, it offers continuous and indefinite monitoring, making it a durable and reliable option for long-term HF management [11].

This article review evaluates the CardioMEMS HF system in terms of clinical efficacy, challenges/limitations to implementation, and future directions.

## Review

Pathophysiology of pulmonary artery and heart failure

The pulmonary artery (PA) is responsible for carrying deoxygenated blood from the right ventricle of the heart into the lungs. Its pressure is regulated by the volume of the blood and the resistance of the vasculature. In HF, there is a compromise in the heart's pumping function, leading to a backup and buildup of pressure within the heart chambers [2]. This increased pressure extends into the pulmonary circulation, causing elevated PA pressures, often defining the pathophysiology of HF [2]. As the left ventricle fails, the fluid backup in the pulmonary veins and increased workload on the right side of the heart eventually contribute to pulmonary hypertension (PH). Elevated pulmonary pressure is a key indicator of worsening HF and an early pointer to fluid overload, laying a foundation for the potential benefits of the CardioMEMS monitoring system [12]. The CardioMEMS sensor is implanted in the pulmonary artery to provide real-time monitoring of pressure changes. This system then transmits these changes wirelessly to healthcare providers, providing a timely alert to impending decompensation and allowing for timely interventions before the overt symptoms of HF such as edema or dyspnea appear [12]. By continuously monitoring the pulmonary artery pressure, clinicians can individually tailor HF management through diuretics and vasodilator therapy precisely, thus reducing hospitalizations for HF [12]. The early identification of and intervention for pulmonary pressure changes can help prevent the progressive remodeling of pulmonary vasculature and right ventricular dysfunction, both of which are pathognomonic of chronic HF [12].

CardioMEMS technology overview

The CardioMEMS system, developed by Abbott, is a groundbreaking implantable microsensor system designed to remotely monitor PA pressure in patients suffering from HF. This advanced technology offers a unique solution to the challenges of managing HF by enabling continuous, real-time monitoring of key hemodynamic parameters, which can help detect early signs of worsening HF before clinical symptoms become apparent [13].

Components of the CardioMEMS System

The CardioMEMS system is composed of three primary components that work in tandem to provide seamless monitoring and data transmission.

Delivery catheter: This specialized catheter is used during the implantation procedure to deliver the wireless microsensor into the pulmonary artery. The catheter is designed to navigate the vascular system, ensuring precise sensor placement in the distal PA under fluoroscopic guidance [13].

Implantable wireless microsensor:The heart of the CardioMEMS system is the wireless microsensor, a small, sophisticated device permanently implanted into the distal PA. The sensor measures approximately 15 × 3.4 × 2 mm and is constructed with a three-dimensional coil (inductor) and a pressure-sensitive capacitor. These components are encapsulated within two slices of fused silica and encased in medical-grade silicone, which enhances the sensor's biocompatibility and durability, ensuring it remains functional for the patient's lifetime [13].

Patient electronics system (PES):The PES is an external device employing radiofrequency technology to wirelessly communicate with the implanted sensor. This system is responsible for collecting, transmitting, and analyzing the pressure data from the sensor, making it accessible to healthcare providers via a secure, user-friendly online platform [13].

Technical Operation of the CardioMEMS Sensor

The CardioMEMS sensor operates on the principles of an inductance-capacitance circuit. The circuit's design allows it to oscillate at a specific resonant frequency when current flows between the inductor and the pressure-sensitive capacitor. The inductor facilitates electromagnetic coupling with the capacitor, enabling the sensor to wirelessly communicate with the external readout device without needing an internal power source, such as a battery [13].

The sensor's initial calibration is performed in the cardiac catheterization laboratory using invasively obtained blood pressure values during catheterization. This calibration process is critical, as it ensures that the sensor provides accurate pressure measurements by converting the detected resonant frequency into precise PA pressure values. The PES then stores and processes these values for ongoing monitoring and analysis [13].

Implantation and Long-Term Functionality

The implantation of the CardioMEMS sensor is a minimally invasive procedure carried out in the cardiac catheterization laboratory. Using a catheter, the sensor is implanted transvenously into a descending branch of either the left or right PA. The procedure is performed under fluoroscopic guidance to ensure accurate placement. Once implanted, the sensor is designed to remain in situ for the patient's lifetime, with no need for leads, generators, or battery replacements, significantly reducing the maintenance burden and risk of complications [13].

Data Transmission and Clinician Monitoring

Post-implantation, the CardioMEMS PA sensor continuously collects PA pressure data, including systolic, diastolic, and mean PA pressures and heart rate. The sensor communicates wirelessly with a pillow-shaped external system (ES) that the patient uses at home to transmit these data to the PES. Patients are instructed to perform daily self-measurements, with the collected data automatically uploaded to a secure online platform. This platform is accessible to clinicians via a web interface, allowing them to monitor a patient's condition in real time [14].

The monitoring system is comprised of several components.

Hospital electronics system (HES):Used primarily during the initial sensor calibration in the hospital, the HES offers advanced functionalities, including real-time display and printing of pressure data. The HES software provides detailed readings of systolic, diastolic, and mean PA pressures, along with pressure waveforms, all displayed on a touchscreen interface [15].

Patient external system (PES):The PES guides patients through acquiring daily PA pressure measurements at home. It automatically uploads the data to the database, accessible to healthcare providers. The PES is designed to be user-friendly, ensuring that patients can easily manage their part of the monitoring process without requiring extensive technical knowledge [14].

Clinical Integration and Decision-Making

The CardioMEMS system is designed to integrate seamlessly into clinical practice, providing clinicians with real-time data that can be used to make informed decisions about heart failure management. Automated notifications are sent to clinicians if pressure readings fall outside predefined ranges or thresholds, prompting timely interventions and medication adjustments. This proactive approach helps to prevent acute decompensation and reduces the need for emergency hospitalizations [13].

Clinicians can use the data provided by the CardioMEMS system to communicate directly with patients, offering personalized guidance and support. By enabling the continuous monitoring and early detection of hemodynamic changes, the CardioMEMS system can significantly improve outcomes for heart failure patients, enhancing both their quality of life and overall survival rates [13].

Clinical evidence and efficacy

Several pivotal trials have established the safety and efficacy of the CardioMEMS HF system across diverse patient populations and clinical settings. Notably, randomized data demonstrate significant benefits specifically in adults with heart failure (HF) symptoms corresponding to New York Heart Association (NYHA) class III or IV, irrespective of HF etiology. However, specific subgroups, such as women and patients with preserved left ventricular ejection fraction (LVEF), were underrepresented in trials such as CardioMEMS Heart Sensor Allows Monitoring of Pressure to Improve Outcomes in NYHA Class III Heart Failure Patients (CHAMPION) and Remote haemodynamic monitoring of pulmonary artery pressures in patients with chronic heart failure (MONITOR-HF) [16].

The CHAMPION trial, a pivotal multicenter, single-blinded randomized controlled trial, rigorously assessed CardioMEMS-guided therapy in reducing HF hospitalizations. A total of 550 NYHA class III HF patients, irrespective of their LVEF and prior HF hospital admission, were enrolled across 64 US sites and were implanted with CardioMEMS. The study's findings were significant, revealing a 37% reduction in HF-related hospitalizations in the treatment group (patients whose readings of PA pressure were disclosed to the treating providers) compared to the control group (where similarly obtained PA pressures were not given to the providers) over a mean follow-up period of 15 months. The trial's results underscore the substantial benefits of hemodynamic-guided therapy in reducing the burden of hospitalizations in HF patients compared to standard care [17].

The Hemodynamic-GUIDEd management of Heart Failure (GUIDE-HF) trial expanded on these findings by enrolling 1,000 patients with NYHA functional classes II-IV, a previous HF hospitalization, or elevated natriuretic peptide levels. Patients were randomized into treatment (hemodynamically guided management) and control arms. The trial demonstrated fewer primary events, including HF hospitalizations (~28%), urgent HF visits, and all-cause mortality at 12 months in the treatment arm compared to the control arm. While this trial had an overall neutral result, it was impacted by the coronavirus pandemic, which resulted in a decrease in hospital admissions in many chronic conditions, including HF. Nevertheless, the pre-pandemic part of the trial showed a significant reduction in morbidity. This trial further highlighted that targeting filling pressures is pivotal in determining HF hospitalization risk across various LVEF subgroups [18].

In real-world settings, the CardioMEMS Post-Approval Study evaluated the effectiveness and safety of the device in 1,200 NYHA class III HF patients. The study confirmed significant reductions in HF hospitalizations, affirming the device's efficacy outside controlled trial environments. Noteworthy, the benefit in the reduction of hospital admissions was even more impressive (a 57% reduction) than in the CHAMPION or GUIDE-HF trial. This evidence supports the broader application of CardioMEMS in diverse clinical settings and patient populations [19].

Similarly, the MONITOR-HF trial in Europe provided additional evidence supporting CardioMEMS use. In this trial, NYHA class III HF patients experienced improved quality of life and reduced hospitalizations (by 44%) when managed with the device compared to those receiving standard care [20]. This study reinforces the role of CardioMEMS in enhancing patient outcomes in a broader international context.

Ongoing trials, such as Pulmonary artery sensor system pressure monitoring to improve heart failure outcomes (PASSPORT-HF), further aim to validate the clinical utility of CardioMEMS in everyday practice. PASSPORT-HF focuses on outcomes such as HF-related re-hospitalizations and mortality in NYHA class III patients under nurse-led care, highlighting the system's adaptability and potential for integration into various care models [21].

Moreover, a meta-analysis of several randomized controlled trials with CardioMEMS demonstrated not only morbidity reduction but also mortality reduction in patients with HF treated with the CardioMEMS guidance [22].

Given these compelling trial results, the American Heart Association (AHA) and American College of Cardiology (ACC) guidelines have assigned a class IIb recommendation for using CardioMEMS in specific adult patients. These patients include those with NYHA class III symptoms and a recent history of HF hospitalizations within the previous year or elevated levels of natriuretic peptides despite maximal tolerated medical and device therapy. This recommendation is designed to mitigate the risk of future HF hospitalizations, emphasizing the importance of precise and proactive HF management strategies [23].

These trials collectively contribute to the growing body of evidence supporting the use of CardioMEMS in improving HF management. They may significantly influence future guideline recommendations and the clinical adoption of this technology.

**Figure 1:**
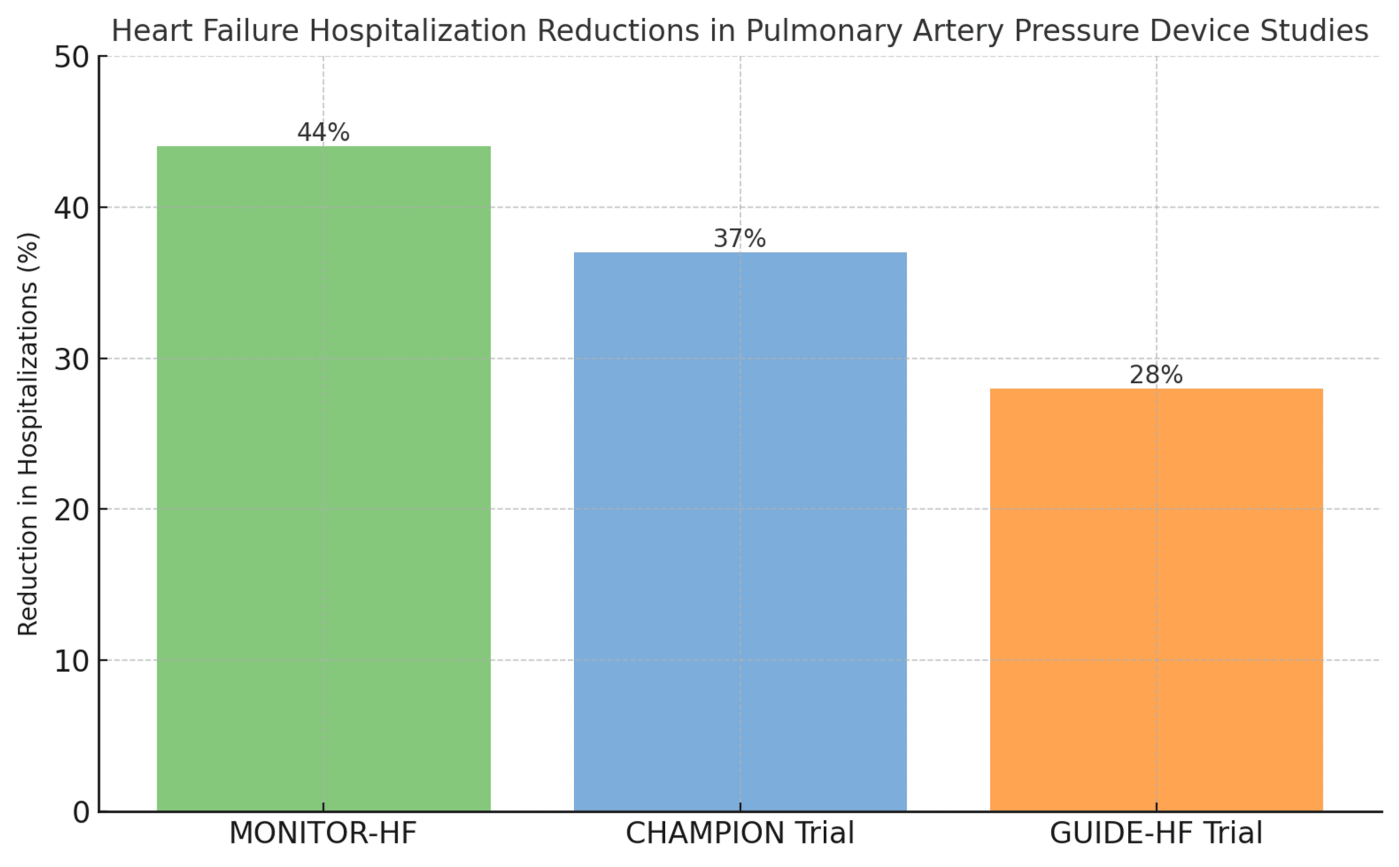
Graphical presentation of percentage reduction in HF hospitalizations among various trials Image created by Oluwaremilekun Tolu-Akinnawo (one of the authors) HF, heart failure; MONITOR-HF, Remote haemodynamic monitoring of pulmonary artery pressures in patients with chronic heart failure; CHAMPION, CardioMEMS Heart Sensor Allows Monitoring of Pressure to Improve Outcomes in NYHA Class III Heart Failure Patients; GUIDE-HF, Hemodynamic-GUIDEd management of Heart Failure

Figure 1 demonstrates the percentage reduction in HF Hospitalizations among various trials.

Integration into heart failure management

HF management is increasingly integrating advanced technologies, with CardioMEMS emerging as a promising tool for monitoring HF progression and optimizing treatment strategies. The CardioMEMS HF system is a notable example, offering unique advantages by continuously tracking key physiological indicators, particularly pulmonary artery pressures, which are critical for assessing and managing HF. These real-time insights enable healthcare providers to make timely and precise adjustments to treatment, potentially preventing acute decompensation and reducing hospitalizations.

In current clinical practice, HF monitoring combines traditional and advanced technologies to enhance patient care. Biomarkers such as B-type natriuretic peptide (BNP) remain fundamental for HF diagnosis and risk assessment, providing insights into cardiac function and fluid status. These biomarkers are often supplemented with outpatient monitoring strategies that include remote symptom tracking via smartphones and applications such as Cardiogram, HeartWatch, and AliveCor's KardiaMobile. Telemonitoring programs using phone calls, video chats, or online platforms also connect patients with healthcare providers, facilitating continuous management and early intervention. However, traditional monitoring methods, while useful, have limitations. For example, implantable technologies such as lung impedance monitors (e.g., Medtronic's OptiVol) assess pulmonary congestion by measuring thoracic impedance changes. These devices are primarily used in patients with implantable pacemakers and defibrillators and have limitations in patient selection and specificity, affecting their overall effectiveness in managing HF.

In contrast, implantable wireless pulmonary artery pressure sensors, such as the CardioMEMS HF system, provide continuous hemodynamic data. While the OptiVol measures the impedance, which results from increased intracardiac pressures (secondary event), CardioMEMS measures the pressure directly (primary event). This capability offers a more comprehensive view of a patient's HF status, enabling early symptom-onset detection and prompt intervention. MEMS sensors excel in remote, real-time data transmission, which is critical for proactive HF management outside clinical settings. This allows healthcare providers to adjust medications promptly and tailor treatment plans based on individual patient responses, potentially reducing the frequency and severity of HF exacerbations. The CardioMEMS HF system is currently the only pulmonary artery pressure sensor approved for routine clinical use by the FDA and the European Conformity (EC) mark. Another device, the Cordella™ Pulmonary Artery Pressure Sensor System (Endotronix, Inc., Naperville, IL), operates on similar hemodynamic principles but remains under investigation and has not received FDA clearance or EC mark [13].

Despite its numerous benefits, MEMS-based monitoring presents several challenges. These include procedural risks during sensor implantation, such as vascular complications, and potential device-related complications such as infection or malfunction. Additionally, the high cost of the device and its implantation can restrict accessibility for specific patient groups, particularly those without adequate insurance coverage or in regions with limited healthcare resources. However, the cost-effectiveness of CardioMEMS remains to be determined, particularly regarding its widespread implementation. While the initial investment in the technology is substantial, the potential savings from reduced hospitalizations and improved patient outcomes could offset these costs in the long term. Further research is needed to assess the financial viability of the CardioMEMS system across different patient populations with varying disease severities and prognoses [24].

Overall, MEMS-based sensors, especially those like the CardioMEMS HF system, represent significant progress in HF care by providing continuous, real-time hemodynamic data essential for personalized treatment strategies. Despite challenges related to sensor implantation and regulatory approvals, the integration of MEMS technology holds great promise for transforming HF management, improving patient outcomes, and reducing healthcare costs associated with HF hospitalizations.

**Figure 2:**
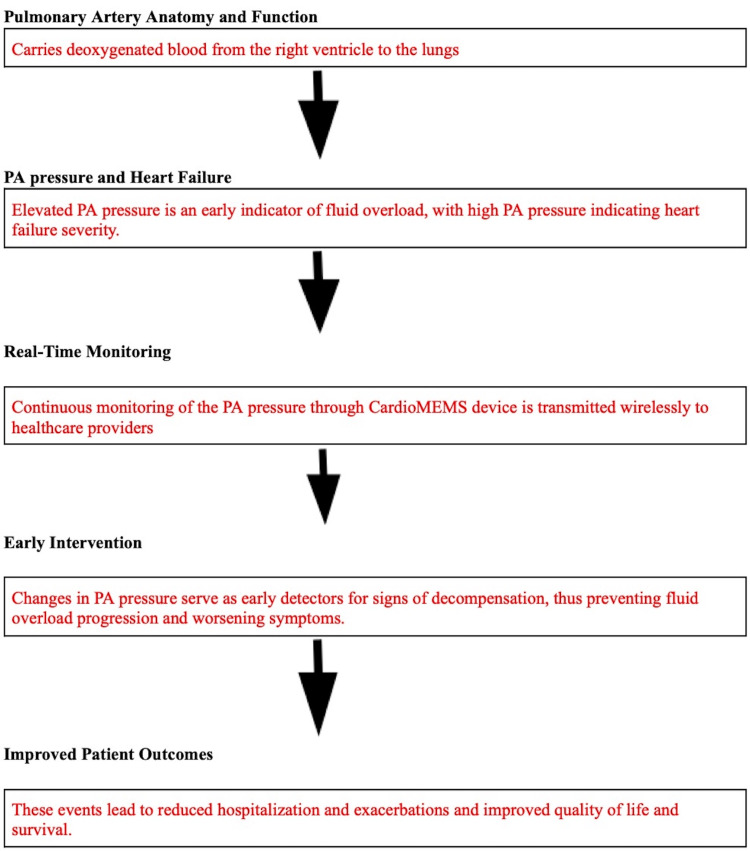
Illustration of the clinical implication of the CardioMEMS sensor on HF management Image created by Oluwaremilekun Tolu-Akinnawo (one of the authors) HF, heart failure; PA, pulmonary artery

Figure 2 illustrates the clinical implication of the CardioMEMS sensor on HF management.

Patient quality of life

Hospital readmissions profoundly negatively impact the quality of life (QOL) and overall patient outcomes. These readmissions escalate healthcare costs and significantly increase the burden on patients and their families. Each subsequent hospital admission raises the likelihood of developing hospital-acquired conditions, such as pneumonia, COVID-19 infection, *Clostridium difficile*, and deep venous thrombosis (DVT) [12]. These conditions can lead to debilitating consequences, further diminishing the QOL of patients [12]. The repetitive nature of hospitalizations exacerbates the risk of deconditioning, which may result in falls and negatively impact mental health, often leading to depression [12,25-27].

For instance, in 2012, 23.4% of patients admitted for decompensated heart failure developed complications such as urinary tract infections (UTIs), pneumonia, or sepsis [25]. These complications not only prolong hospital stays but also increase healthcare costs and burden the healthcare system while further complicating the management of heart failure (HF) patients. Another study found that among patients aged 65 years and older, 61.55% of 4,754 falls resulted in femur fractures, which severely reduced independence and further worsened QOL [26].

Depression is another critical factor that profoundly affects QOL, length of hospital stays, and post-discharge mortality [27]. It is estimated that 35%-60% of patients with decompensated heart failure experience depression [27]. Given that CardioMEMS has been shown to reduce the risk of heart failure decompensation and subsequent hospital readmissions, its use could potentially mitigate these complications and enhance the QOL of heart failure patients.

Moreover, recurrent hospital readmissions disrupt patients' and caregivers' lives, taking them away from their families, work, and other essential activities. This disruption worsens QOL and diminishes economic productivity and financial stability, both of which are associated with poorer health outcomes. Additionally, hospital readmissions are linked with increased mortality rates among heart failure patients [12]. Indeed, contemporary research has identified a greater than 75% increase in five-year mortality following the first hospitalization for heart failure [12].

Economic impact

According to a recent survey, the treatment of heart failure incurs over $20.9 billion in total healthcare expenditures, underscoring its significance as a major public health concern [28]. A substantial portion of these costs is attributed to the treatment of decompensated heart failure, which leads to at least one million hospital admissions annually [28,29]. The CHAMPION trial demonstrated clear benefits of using CardioMEMS, including fewer hospitalizations among the treatment group and improved quality of life. A recent study employing a Markov model to evaluate hospitalization rates, survival, quality of life, costs, and the incremental cost-effectiveness ratio (ICER) of CardioMEMS implantation versus standard care among CHAMPION trial candidates with heart failure identified significant cost-effectiveness benefits [30]. The study found that CardioMEMS led to a reduction in lifetime hospitalizations (2.18 versus 3.12) and an increase in quality-adjusted life years (QALYs) (2.74 versus 2.45). However, this was accompanied by a slight cost increase ($176,648 versus $156,569), resulting in a cost of $71,462 per QALY gained and $48,054 per life year gained [30]. Additionally, the cost per QALY gained was $82,301 in patients with heart failure with reduced ejection fraction (HFrEF) and $47,768 in those with HF with preserved ejection fraction (HFpEF) [30]. In the lower-risk Candesartan in Heart failure: Reduction in Mortality and Morbidity (CHARM) cohort, it was estimated that the device would need to reduce hospitalizations by 41% to achieve a cost-effectiveness threshold of less than $100,000 per QALY gained, highlighting the sensitivity of cost-effectiveness to the device's durability [24]. Similarly, in the Netherlands, a randomized controlled trial recently confirmed reduced HF hospitalizations and improved quality of life with the hemodynamic monitoring of pulmonary pressures. In the United Kingdom, a cost-utility analysis study noted that the CardioMEMS HF system was an effective strategy for HF patients, further validating its use in diverse healthcare settings [20,31].

Challenges and limitations

The CardioMEMS HF system is undeniably one of the most significant innovations in managing heart failure (HF), offering notable benefits such as reduced HF readmissions and improved patient outcomes. However, despite its advantages, several challenges and limitations warrant careful consideration.

Safety Concerns

Reports from the initial years of CardioMEMS use in the United States highlight a relatively low rate of adverse events, including sensor-related complications and vascular access issues, indicating the device's favorable safety profile [32]. Similar European experiences affirm the feasibility and safety of tailored treatment based on PAP measurements within different healthcare systems [33,34]. For these reasons, the safety profile of the CardioMEMS HF system has been scrutinized, particularly through data from the Manufacturer and User Facility Device Experience (MAUDE) database, a passive surveillance system that collects both mandatory and voluntary reports of device-related malfunctions, injuries, or deaths submitted to the FDA. A recent study analyzed data from May 28, 2014, the date of FDA premarket approval of the CardioMEMS system, to evaluate its safety [32]. During the first three years post-approval, more than 5,500 CardioMEMS devices were implanted across the United States, with at least 155 unique adverse events reported, accounting for 2.8% of the total implants [32].

Of these 155 adverse events, 94.8% (147) were mandatorily reported by the manufacturer or user facilities. The reported adverse events accumulated gradually over time, although some, particularly those related to pulmonary artery (PA) injury, hemoptysis, and even death, occurred immediately post-implantation [27]. Specifically, there were 28 cases of PA injury/hemoptysis, of which 14 required intensive care unit (ICU) admission, seven necessitated intubation, and six resulted in death [32]. Additionally, sensor failure, device malfunction, and device migration were reported in 46 cases. Of these 46 cases, 35 required recalibration, 13 necessitated reimplantation, and 11 resulted in hospitalization. Notably, five sensors could not be used despite recalibration attempts [32]. Other complications included access site-related bleeding or infection in 15 cases and pulmonary embolism (PE) or device thrombosis in five cases [27]. The MAUDE database also recorded 22 deaths, with six directly attributable to PA injury/hemoptysis, four related to HF, and 12 unknown causes, which may or may not have been related to the device [32]. It is important to note that the quality of this report was limited by the scant patient history details provided and the absence of autopsies.

Another significant safety concern is the potential interference of the CardioMEMS device with other medical devices, such as pacemakers, cardiac defibrillators, magnetic resonance imaging (MRI) machines, and implantable neurostimulators. This interference raises substantial safety concerns and could complicate diagnostic procedures when these devices are needed.

Patient Suitability

Patients' suitability for CardioMEMS implantation is another limitation, as certain comorbidities may contraindicate its use. For instance, patients with active infections, a history of pulmonary embolism (PE) or deep vein thrombosis, congenital heart disease, or indwelling mechanical right heart valves may not be suitable candidates for CardioMEMS implantation. Additionally, patients who cannot tolerate a right heart catheterization, which is a necessary procedure for device placement, are also unsuitable [32]. Coagulation disorders pose another challenge due to the increased risk of bleeding or thrombosis, especially given the need for antiplatelet therapy with aspirin or clopidogrel post-implantation, which are essential to preventing thrombotic complications [32].

Moreover, monitoring the device is particularly challenging in patients with advanced chronic kidney disease, limiting its applicability in this patient population.

Cost Considerations

While the implementation of remote hemodynamic monitoring offers remarkable benefits through the reduction of HF-related hospitalizations, one of the most significant barriers to the widespread implementation of the CardioMEMS HF system is its cost. Despite the system's demonstrated ability to improve mortality rates, reduce hospitalizations, enhance quality-adjusted life years (QALYs), and achieve a favorable incremental cost-effectiveness ratio, the initial cost of the device remains a formidable obstacle. The CardioMEMS system is considerably more expensive than the standard of care (SoC) across several countries, with costs ranging from $201,437 in the United States to $25,963 in the United Kingdom [35].

These high costs raise important questions about the accessibility of the CardioMEMS system for uninsured patients and those from lower socioeconomic backgrounds. The financial barrier to implementation could exacerbate existing health disparities, making it less likely that these vulnerable populations will benefit from this advanced technology. This could contribute to a worsening trend in health outcomes for those already disadvantaged.

Invasiveness

This is a key concern. Although the device is small and minimally invasive, a surgical procedure is still needed to implant the sensor in the pulmonary artery. This aspect may raise safety concerns for some patients, as well as limit the suitability for those who may not tolerate the procedure well.

Dependence on External Systems

Although the CardioMEMS device collects pressure readings from the heart, patients must rely on external electronics to transmit and monitor the data. Any break in this transmission that may be secondary to technical failures, connectivity issues, or system maintenance needs can disrupt this flow of vital information, potentially reducing the effectiveness of the CardioMEMS device.

Limited Data Analysis

The CardioMEMS device focuses mainly on a single type of data, which means that it does not provide insights into other important factors such as heart rhythm or oxygen levels. This is due mainly to its ability to analyze only a narrow set of information, thus missing signs of other changes in the patient's condition.

Future directions

The evolving world of heart failure (HF) management, particularly in patients with heart failure with reduced ejection fraction (HFrEF), continues to necessitate innovative approaches that go beyond conventional methods. The CardioMEMS HF system has proven to be one of the most valuable innovations in the recent world in reducing re-hospitalizations and improving patient outcomes through continuous, remote monitoring of pulmonary artery pressures. However, several key areas require further exploration and development to optimize its potential and widespread implementation.

Expanding Indications and Patient Selection

While current evidence supports the use of CardioMEMS in patients with NYHA class III symptoms, there is a growing interest in exploring its application in other patient subgroups. Future research should focus on expanding patient selection and widespread adoptions with consideration to include patients with NYHA class II symptoms, those with HFpEF, and individuals with comorbid conditions particularly chronic kidney disease, which pose poor patient outcomes in patients with coexisting HF. Also, investigating the system's efficacy in pediatric and geriatric populations could broaden its utility and prove beneficial. Besides, there may be benefits in utilizing CardioMEMS in adults with congenital heart disease, patients on hemodialysis, and primary pulmonary hypertension. The idea of combining CardioMEMS with a left ventricular assist device, where the speed would be automatically adjusted based on PA pressures, a so-called "smart pump" concept, is also attractive [36].

Integration With Advanced Analytics and Artificial Intelligence (AI)

With the continued adoption and integration of artificial intelligence (AI) into clinical practice, the integration of CardioMEMS with advanced analytics, including machine learning algorithms and AI, presents a promising avenue for enhancing its predictive capabilities. AI-driven models could analyze the vast amounts of data generated by the CardioMEMS device to identify subtle trends and patterns that may precede clinical deterioration, allowing for even earlier intervention than we currently have. Additionally, incorporating predictive analytics into the system could aid in personalizing treatment plans based on individual patient profiles, optimizing outcomes [37].

Cost-Effectiveness and Accessibility

Despite the obvious clinical benefits of CardioMEMS, its high cost continues to remain a barrier to widespread adoption, particularly in resource-limited settings and low-income earners further bridging health disparities. Future studies should focus on conducting comprehensive cost-effectiveness analyses in various healthcare settings to determine the long-term economic impact of the system. Additionally, efforts to reduce manufacturing and implantation costs, coupled with exploring alternative financing models, could make this technology more accessible to a broader patient population, hence potentially reducing health disparity.

Enhancing Patient and Provider Engagement

The effective use of the CardioMEMS system relies heavily on patient adherence to daily monitoring routines and proactive engagement from healthcare providers. Future initiatives should explore ways to enhance patient education, simplify the user interface of the monitoring system, and integrate behavioral health support to address any psychological barriers to consistent use. Moreover, developing more intuitive platforms for clinicians that streamline data interpretation and clinical decision-making could improve HF management's overall efficiency and effectiveness.

Long-Term Outcomes and Real-World Evidence

While randomized controlled trials have demonstrated the efficacy of CardioMEMS, long-term data on its impact on mortality, quality of life, and healthcare resource utilization are still limited. Ongoing and future studies should aim to collect and analyze long-term outcomes in diverse patient populations and real-world settings. Additionally, establishing robust registries to track post-market outcomes and adverse events could provide valuable insights into the system's performance over time.

Global Implementation and Regulatory Approvals

For CardioMEMS to achieve global impact, it is essential to navigate the complexities of regulatory approvals and adoption across different healthcare systems. Future efforts should focus on obtaining regulatory clearances in regions where the system still needs approval, such as parts of Asia and Africa. Collaborating with international health organizations and local governments could facilitate the implementation of CardioMEMS in these regions, ensuring that its benefits are available to a broader array of patients worldwide.

Innovation in Sensor Technology

Continuous innovation in sensor technology is critical for the future success of CardioMEMS. Research into improving sensor accuracy, reducing the device's size, and extending its durability could further enhance its clinical utility. Moreover, developing multi-sensor platforms that can monitor additional hemodynamic parameters or integrate with other implantable devices may offer more comprehensive management solutions for patients with complex cardiovascular conditions.

## Conclusions

The CardioMEMS HF system marks a transformative advancement in heart failure management, particularly for patients with reduced ejection fraction. By providing continuous hemodynamic monitoring, this technology enables the earlier detection of HF exacerbations, allowing for timely therapeutic adjustments that can significantly reduce hospitalizations and improve patient quality of life. Clinical trials and real-world studies have validated its efficacy and safety, making it a valuable tool in contemporary heart failure care. However, challenges such as procedural risks, high costs, and patient selection criteria must be carefully navigated to optimize its broader application. As healthcare systems evolve, integrating such advanced monitoring technologies will be crucial in addressing the growing burden of heart failure, improving outcomes, and reducing the economic strain associated with this prevalent condition. Further research is needed to refine the cost-effectiveness of CardioMEMS, ensuring its benefits are accessible to a broader range of patients in varying healthcare environments.

## References

[1] Sicras-Mainar A, Sicras-Navarro A, Palacios B, Varela L, Delgado JF (2022). Epidemiology and treatment of heart failure in Spain: the HF-PATHWAYS study. Rev Esp Cardiol (Engl Ed).

[2] Patel J, Rassekh N, Fonarow GC, Deedwania P, Sheikh FH, Ahmed A, Lam PH (2023). Guideline-directed medical therapy for the treatment of heart failure with reduced ejection fraction. Drugs.

[3] Ponikowski P, Voors AA, Anker SD (2016). 2016 ESC guidelines for the diagnosis and treatment of acute and chronic heart failure. Rev Esp Cardiol (Engl Ed).

[4] Brouwers FP, de Boer RA, van der Harst P (2013). Incidence and epidemiology of new onset heart failure with preserved vs. reduced ejection fraction in a community-based cohort: 11-year follow-up of PREVEND. Eur Heart J.

[5] Conrad N, Judge A, Tran J (2018). Temporal trends and patterns in heart failure incidence: a population-based study of 4 million individuals. Lancet.

[6] Roger VL (2021). Epidemiology of heart failure: a contemporary perspective. Circ Res.

[7] Escobar C, Varela L, Palacios B (2020). Costs and healthcare utilisation of patients with heart failure in Spain. BMC Health Serv Res.

[8] Maggioni AP, Dahlström U, Filippatos G (2013). EURObservational Research Programme: regional differences and 1-year follow-up results of the heart failure pilot survey (ESC-HF Pilot). Eur J Heart Fail.

[9] Ciotola F, Pyxaras S, Rittger H, Buia V (2024). MEMS technology in cardiology: advancements and applications in heart failure management focusing on the CardioMEMS device. Sensors (Basel).

[10] Radhoe SP, Clephas PR, Mokri H, Brugts JJ (2023). The CardioMEMS heart failure system for chronic heart failure - a European perspective. Expert Rev Med Devices.

[11] Codina P, Vicente Gómez JÁ, Hernández Guillamet G (2024). Assessing the impact of haemodynamic monitoring with CardioMEMS on heart failure patients: a cost-benefit analysis. ESC Heart Fail.

[12] Joshi R, Nair A (2021). The utility of CardioMEMS, a wireless hemodynamic monitoring system in reducing heart failure related hospital readmissions. J Nurse Pract.

[13] Scholte NT, van Ravensberg AE, Shakoor A (2024). A scoping review on advancements in noninvasive wearable technology for heart failure management. NPJ Digit Med.

[14] (2024). CardioMEMS implantation procedure overview. https://www.cardiovascular.abbott/us/en/hcp/products/heart-failure/pulmonary-pressure-monitors/cardiomems/procedure-overview.html.

[15] Shavelle D, Jermyn R (2016). The CardioMEMS heart failure sensor: a procedural guide for implanting physicians. J Invasive Cardiol.

[16] DeFilippis EM, Henderson J, Axsom KM (2021). Remote hemodynamic monitoring equally reduces heart failure hospitalizations in women and men in clinical practice: a sex-specific analysis of the CardioMEMS Post-Approval Study. Circ Heart Fail.

[17] Abraham WT, Adamson PB, Bourge RC (2011). Wireless pulmonary artery haemodynamic monitoring in chronic heart failure: a randomised controlled trial. Lancet.

[18] Zile MR, Mehra MR, Ducharme A (2022). Hemodynamically-guided management of heart failure across the ejection fraction spectrum: the GUIDE-HF Trial. JACC Heart Fail.

[19] Shavelle DM, Desai AS, Abraham WT (2020). Lower rates of heart failure and all-cause hospitalizations during pulmonary artery pressure-guided therapy for ambulatory heart failure: one-year outcomes from the CardioMEMS Post-Approval Study. Circ Heart Fail.

[20] Brugts JJ, Radhoe SP, Clephas PR (2023). Remote haemodynamic monitoring of pulmonary artery pressures in patients with chronic heart failure (MONITOR-HF): a randomised clinical trial. Lancet.

[21] Störk S, Bernhardt A, Böhm M (2022). Pulmonary artery sensor system pressure monitoring to improve heart failure outcomes (PASSPORT-HF): rationale and design of the PASSPORT-HF multicenter randomized clinical trial. Clin Res Cardiol.

[22] Lindenfeld J, Costanzo MR, Zile MR (2024). Implantable hemodynamic monitors improve survival in patients with heart failure and reduced ejection fraction. J Am Coll Cardiol.

[23] Writing Committee Members, ACC/AHA Joint Committee Members (2022). 2022 AHA/ACC/HFSA guideline for the management of heart failure. J Card Fail.

[24] Sandhu AT, Goldhaber-Fiebert JD, Owens DK, Turakhia MP, Kaiser DW, Heidenreich PA (2016). Cost-effectiveness of implantable pulmonary artery pressure monitoring in chronic heart failure. JACC Heart Fail.

[25] Tu JV, Nardi L, Fang J, Liu J, Khalid L, Johansen H (2009). National trends in rates of death and hospital admissions related to acute myocardial infarction, heart failure and stroke, 1994-2004. CMAJ.

[26] Nieminen MS, Dickstein K, Fonseca C (2015). The patient perspective: quality of life in advanced heart failure with frequent hospitalisations. Int J Cardiol.

[27] McManus DD, Piacentine SM, Lessard D, Gore JM, Yarzebski J, Spencer FA, Goldberg RJ (2011). Thirty-year (1975 to 2005) trends in the incidence rates, clinical features, treatment practices, and short-term outcomes of patients <55 years of age hospitalized with an initial acute myocardial infarction. Am J Cardiol.

[28] Mozaffarian D, Benjamin EJ, Go AS (2015). Heart disease and stroke statistics--2015 update: a report from the American Heart Association. Circulation.

[29] Dunlay SM, Shah ND, Shi Q, Morlan B, VanHouten H, Long KH, Roger VL (2011). Lifetime costs of medical care after heart failure diagnosis. Circ Cardiovasc Qual Outcomes.

[30] Cowie MR, Thokala P, Ihara Z, Adamson PB, Angermann C (2023). Real-time pulmonary artery pressure monitoring in heart failure patients: an updated cost-effectiveness analysis. ESC Heart Fail.

[31] Cowie MR, Simon M, Klein L, Thokala P (2017). The cost-effectiveness of real-time pulmonary artery pressure monitoring in heart failure patients: a European perspective. Eur J Heart Fail.

[32] Vaduganathan M, DeFilippis EM, Fonarow GC, Butler J, Mehra MR (2017). Postmarketing adverse events related to the CardioMEMS HF system. JAMA Cardiol.

[33] Angermann CE, Assmus B, Anker SD (2020). Pulmonary artery pressure-guided therapy in ambulatory patients with symptomatic heart failure: the CardioMEMS European Monitoring Study for Heart Failure (MEMS-HF). Eur J Heart Fail.

[34] Cowie MR, Flett A, Cowburn P (2022). Real-world evidence in a national health service: results of the UK CardioMEMS HF System Post-Market Study. ESC Heart Fail.

[35] Schmier JK, Ong KL, Fonarow GC (2017). Cost-effectiveness of remote cardiac monitoring with the CardioMEMS heart failure system. Clin Cardiol.

[36] Thohan V, Abraham J, Burdorf A (2023). Use of a pulmonary artery pressure sensor to manage patients with left ventricular assist devices. Circ Heart Fail.

[37] Dixon D, Sattar H, Moros N (2024). Unveiling the influence of AI predictive analytics on patient outcomes: a comprehensive narrative review. Cureus.

